# Knowledge and Practices of In-Home Pesticide Use: A Community Survey in Uganda

**DOI:** 10.1155/2011/230894

**Published:** 2011-06-05

**Authors:** Eva Nalwanga, John C. Ssempebwa

**Affiliations:** School of Public Health, College of Health Sciences, Makerere University, P.O. Box 7072, Kampala, Uganda

## Abstract

Many communities in low-income countries use in-home pesticides for the control of pests. Such use is often inadequately controlled. In this study, 100 households in Kireka ward, Wakiso district in Uganda were involved in a cross-sectional survey to assess pests, knowledge, and use patterns of pesticides. A structured pretested questionnaire was administered via personal interviews, and observational checklists were used. Mosquitoes were the most prevalent pests (83%), followed by cockroaches (69%) and rats (52%). Pesticides were the most preferred method for pest control (98%), with insecticide spray being the most common form of application (71.4%). Pesticide application was inappropriately done in many households mainly due to inadequate knowledge on use. Only 48% of the respondents read manufacturer's instructions for use. Information on what pesticide to use was obtained from friends (53.1%), points of sales (48%). Educational interventions particularly at points of sale would be a critical avenue for promoting safe use of pesticides in households.

## 1. Introduction

There has been considerable research on the use and exposure to pesticides in agricultural operations in low-income countries [1–6]. Most of the research has been based in rural settings where agriculture is done [7]. Less information is available about the use of pesticides in households within periurban settlements in low-income countries [8]. 

Periurban settlements in low-income countries are characterized by inadequate environmental sanitation. Water, sanitation, and hygiene have been frequently addressed in these settings, often leaving health conditions or risks associated with vectors and vermin overlooked and not well integrated into public health policies. Specific urban risks and causes of mortality and morbidity have been associated with presence of pests such as insects and rodents [9, 10]. 

Good-quality housing is a key element for ensuring healthy communities, and this poses a challenge in periurban areas. Poor housing gives rise to poor hygiene and results in health problems such as bedbugs, cockroaches, mosquitoes, fleas, lice, rats, and other vectors [11]. Poor drainage of household wastewater and rain water causes a number of environmental and health hazards, for example, formation of stagnant pools of water that provides breeding sites for mosquitoes. Uncollected refuse provide breeding grounds for disease vectors, such as cockroaches, flies, and rats. The most common approach used to control these pests in enclosed environments like in homes is use of pesticides, particularly insecticides and rodenticides. Poor use and storage of pesticides especially in poor urban settings has contributed to the health burden in these settings [12]. 

The home environment is widely considered to be the most common pesticide-treated indoor environment, where the residents themselves apply the pesticides [13]. Pesticide use contributes to indoor contamination and has the potential to cause human poisoning that are major environmental and health challenges [14, 15]. In South Africa, DDT was detected in all the media analyzed after its use for indoor residual spraying to control malaria vectors [16, 17]. 

Exposure to pesticides has been associated with many health effects that include immediate effects like headaches and nausea [18], delayed effects like intrauterine growth retardation [19], immune toxicity [20], birth defects [21], cancers [22, 23], nervous system disorders [24], and allergic effects like Asthma [25], skin and eye irritations [26], depression [27], and childhood leukaemia [28]. Some of these have been reported with residential exposures, whereas others are potential ones when pesticides are inappropriately used in residential settings.

Kireka ward being a periurban area is faced with several environmental challenges, among which are pests. During the control of these pests, the community could be predisposing itself to health risks due to inappropriate handling and use of pesticides. The purpose of this study was to determine the types of pests in homes of Kireka ward and to assess community knowledge of the dangers and the practice of pesticide use within homes for pest control. The findings have the potential to provide useful information on this so often neglected source of hazardous materials within households in low-income countries, so that effective strategies to increase awareness regarding the use of pesticides in urban communities can be developed. Presently there are no studies detailing the use of pesticides in homes in Uganda to compare with the findings of this study.

## 2. Methods

### 2.1. Study Area

The study was conducted in Kireka ward, one of the six wards in Kira town council, Wakiso district approximately six kilometres from Kampala, the capital city of Uganda. The study area has a total population of 54,009 people, 4,861 households, and a mean household size of 4.2 [29]. Kireka ward has a total of nine zones which are Kireka A, Kireka B, Kireka C, Kireka D, Kamuli A, Kamuli B, Kamuli C, Kasokoso, and Nalya. The site has a rapid population growth due to migration of people from rural to urban centres, which has led to congestion in the area. The type of housing in the study area is formal. Poor garbage disposal, poor drainage, and unkempt bushes have contributed to pest infestations in the area. Within the area, there are pesticide vendors, private vermin, and vector control personnel. The pest management in the study area is supervised by a health Inspector, who is supposed to conduct education on pest control measures and to overseer the activities of private pest controllers.

### 2.2. Study Design

The study was cross-sectional in nature and employed quantitative methods of data collection. Research assistants administered structured questionnaires and also used observational check lists. Training of research assistants was done to ensure their capacity to collect the required information. The questionnaire was translated into Luganda; the language used in the study area, and was pretested to establish its adequacy in obtaining the required information. The study population consisted of all households in the community of Kireka ward.

Previsits to the study area were made to discuss the exercise with the relevant authorities, and permission was sought from the town council authorities. The purpose of the study was explained to all the respondents, and their consent was obtained prior to administering the questionnaires, and only codes were used. The entire exercise was supervised by the Principal Investigator.

### 2.3. Study Participants

The sample size was 100 households, determined using the Leslie Kish formulae [30], 1965. Participants were selected using systematic sampling from four purposively selected zones according to known pest infestation: Kireka A, Kireka B, Kamuli D, and Nalya out of the nine zones in the ward. The number of households participating per zone was proportionate to the total number of households in a particular zone. The study participants were household heads or any adult household member who was present in the home at time of the study. Approximately only 25 of the respondents were household heads. Additional information was obtained from purposively selected key informants, who were pesticide vendors, vermin and vector control personnel, and the health inspector.

### 2.4. Statistical Analysis

Data and information collected were coded, entered, and analyzed using Epi Info statistical analysis package version 6.0, and descriptive statistics were calculated.

## 3. Results and Discussion

Of the 100 respondents, 71% were female. The largest proportion (46%) was married. Other demographic characteristics of the respondents are shown in Table 1. Most of the respondents (53%) had attained secondary education, which would indicate a good level of literacy among the community, and hence they were able to read and understand instructions for use of the pesticides. 

### 3.1. Common Pests in Homes of Kireka Ward

Mosquitoes were the most prevalent pests reported (83%), followed by cockroaches (69%) and rats (52%). Others are indicated in Table 2. Community observations indicated significant stagnant water in the community, resulting from indiscriminate disposal of wastewater, blockages of drainage channels due to obstructions and also siltation, creating good breeding habitats for the malaria vectors. Poor garbage disposal, poor drainage, unkempt bushy areas, and favourable climatic conditions for breeding contribute to the pest infestation in the area. 

In many households, plates and dishes are kept unwashed overnight inside the houses and are washed the following day. This means that food remains are kept inside the house overnight and consequently act as good sources of food for pests like rats and cockroaches. Cockroaches have been implicated with causing disease in households [31]. Cockroach and rodent allergens are associated with morbidity and mortality of asthmatics [32]. 

In five households, animals such as rabbits, chicken, and ducks were being kept inside houses at night. The practice of humans living in close proximity to animals is a significant contributor to pests within urban areas [33]. It has been reported that these animals usually harbour pests like fleas and lice [34–36].

### 3.2. Pest Control Methods Practiced in Kireka Ward

The majority of respondents 98% indicated that households used pesticides, more than any other methods available for pest control (Table 3). They indicated that pesticides were convenient to use, and they also believed them to be more effective and faster at killing the pests than other control methods. Integrated pest management control methods were mentioned as some of those are being employed. These included biological control that involves use of cats for rat control (20%), proper garbage storage and disposal (34%), and use of rat glue and rat traps were also mentioned (22%). In all the 20 households that adopted the biological control as one of the controls, they reported it being a safer alternative especially in households with children. Of the 34 households that did proper garbage storage and disposal, 24 indicated that most times the food remains were given to cats and/or dogs. 

The town council has vector and vermin control officers whose work is to conduct surveillance and to guide the control measures of all pests of human importance within the town council. However, their work is biased towards public places, for example, restaurants and markets and not much is being done at household level. Most pest control services at household level are performed by licensed private pest control personnel whose activities are overseen by the town vector and vermin control officer. Because professional pest control services are perceived as expensive by the community, most households manage pests as they see fit. In all circumstances the costs involved determine the option adopted. At household level, the pest control personnel mainly engage in mosquito and rat control, where for mosquitoes they commonly use cyhalothrin, and for rats they use the rodenticide zinc phosphide. Zinc phosphide has been reported to result in poisonings, and, therefore, requires professional application [37, 38]. 

It was also reported that Indomethacin, a nonsteroidal anti-inflammatory drug, was being used as a rat poison, and it had been found to be very effective and a cheaper alternative. The practice was to mix two 50 mg capsules of indomethacin into approximately 100 gm of food that has a strong fish or meat flavour, often left over from a meal. Putting more than two capsules into the food often results in the rats not eating the bait. The rats start dying after 2-3 days. However, it is not clear whether the rats eat the bait once or more than once before death occurs. 

In humans, indomethacin inhibits the synthesis of prostaglandins, and, therefore, is effective in relieving pain, swelling, and other symptoms and signs of inflammation [39]. The mechanism by which indomethacin causes death in rats could be through phospholipase A2 activation, which either enhances inhibitory synaptic transmission in rat substantia gelatinosa neurons, which play a pivotal role in regulating nociceptive transmission in the spinal cord [40], or it gets involved in the damage of enterocytes in the rat small intestine [41], or gastric damage via ulcerations [42]. Omatsu and colleagues [43] have reported indomethacin-induced small intestinal mucosal injury through mechanisms of apoptosis, induction of reactive oxygen species production and an increase in the protein S oxidation in a rat intestinal epithelial cell line (RIE-1).

Among the 98 respondents that indicated use of pesticides in their households, 71.4% used them in spray form, wettable powders (41.8%), insecticide chalk (39.8%), coils (38.8%), and powder and concentrates (4.1%) (Table 3). The product and brand names were identified from recall and also observations in the household. Use of liquid concentrates was low, and the common one was diazinon, which was found to be most effective on bedbugs. Diazinon, which has been banned in many developed countries, causes high acute toxicity to a wide variety of animals, leading to a wide range of sublethal biochemical effects, damage to specific target organs and tissues, cytotoxic and genotoxic effects, reproductive damage, and adverse ecological impacts [44]. It has great potential for negatively affecting nontarget organisms [45]. For powders, it was zinc phosphide, which was used and was only handled by the technical personnel.

There was a general response that rodenticides were generally not affordable by many households and were no longer effective in killing the rats. All sprays used contained pyrethroids, for example, cypermethrin, deltamethrin, permethrin, and pyrethrin formulations. All sprays were known by brand name, for example, Mortein Doom, Bop, Kilit, Baygon, Ridsect, and Farco insecticides. Pyrethroids have the advantages that they quickly break down in the environment and have a very low toxicity to mammals and humans, but may have chronic effects, for example, endocrine disruption [46, 47].

Wettable powders used include cyhalothrin, deltamethrin k-Othrine, and permethrin. These were mostly used for retreatment of mosquito nets. Wettable powders require a skilled person to apply them. Insecticide chalks were used to kill cockroaches and ants, and their active ingredient was deltamethrin. Insecticide chalks were not as much used as the sprays, which were perceived to be more effective at killing the target pests. Mosquito coils were also common, and the active ingredient was deltamethrin, which combines a long residual effect with quick killing after only brief contact [48]. 

Uganda's malaria control programme has piloted indoor residual DDT spraying as part of its control methods. Although this has not been conducted in the study area, it would have significant implications on the domestic pesticide applications mainly because of its long-term residual effects [49, 50].

### 3.3. Sources of Information on Insecticide Usage

The sources of information about which form and type of insecticide to use in a household included radio and television advertisements, sales points, health inspectors, and from friends as seen in Table 3. The majority of responses among the 98 respondents that indicated the use of pesticides, 53.1%, indicated that many people relied on friends, who included relatives and neighbours for guidance on choice of insecticide to use. Such information draws on prior positive experience of using the insecticide by the person giving the information. Grey and colleagues in their study in the UK showed that friends and relatives had a contributory role in the choice of insecticides [51]. 

Advertisements (33.7%) on radio and television were more relied upon than the community health inspectors (24.5%) as a source of information for pesticide use. It is the role of the community health inspectors to advise on vector and vermin management practices during home visits and community inspections. Advertisements especially over radio should be utilized more in disseminating information about pests and pesticide use by the ministry of health.

Points of sales, both retail and farm supply shops, were a significant source of information and advice, 48%. Retail and farm supply shops are an important point of contact between the seller and users of the pesticides and, therefore, would be a vital interface to convey proper use information that should be more utilized by health authorities. Emphasis on sensitizing the community on in-home pesticide application should be made by the health inspectors since the study indicated that these were the least contributor of information.

### 3.4. Location of Insecticide Application within Households

Pest control operations within a household were the responsibility of the adults. Among the respondents that used pesticides, 73.5% indicated that insecticides got applied directly on house walls. These included wettable powders such as cyhalothrin, insecticide chalks that contained active ingredients of deltamethrin 7.5 g/kg and cypermethrin 1.0% w/w. In 43% of households, applications were made directly on the pest when seen crawling, flying, or resting on walls. Improper application of pesticides exists because quite a number of respondents practiced aerial application or directly on the pest. Although such applications may kill the pests, in aerial spraying it is likely that some fraction of the pesticide ends up on the floor and consequently into dust. 

House dust is a sink and repository for semivolatile organic compounds and particle-bound matter, and several studies have identified house dust as an important source of toxicant exposure [52–54]. Pesticides applied outside or within the household that are absorbed and preserved by house dust can lead through the everyday activities of children and infants to increased exposure [53]. Review of the literature points out that ingestion of house dust may be a major route of exposure to pesticides for infants and toddlers [14]. 

### 3.5. Observation of Manufacturer's Instructions for Use and Efficiency during Application of Insecticides within Households

#### 3.5.1. Reading of Manufacturers Instructions

**Figure 1:**
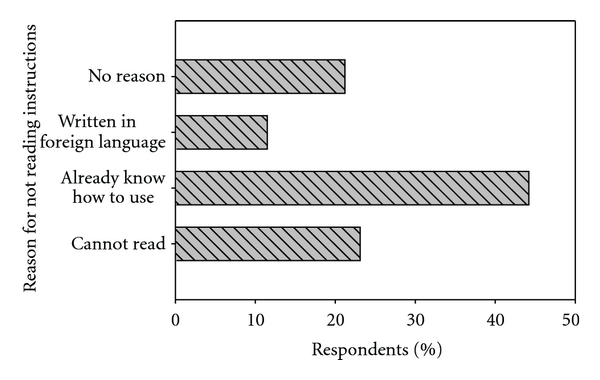
Reasons for not reading instructions for pesticide application.

The manner in which a pesticide product is used is likely to be driven by the extent of knowledge an individual has on the product [55]. Only 48% of the respondents indicated that they read manufacturer's instructions before applying insecticides in their households. The reasons advanced for not reading were as indicated in Figure 1. Those who had used the insecticide before (44.2%) felt no need to read the instructions every time they purchased the product. This has health implications in that, if there is a change in the formulation or concentration, such users would still apply the insecticides following inappropriate application methods. Those that did not read because of illiteracy were 23.1%, while 11.5% were literate but could not read nor understand the foreign languages in which the instructions were written. Languages contained on the products included Chinese, Swahili, and Arabic.

The pesticide label is often the only access users have to pesticide-risk information. The foreign languages used on some pesticides make it difficult for users who do not understand the language to read and understand the instructions before use and consequently this leads into misuse of pesticides [56]. Foreign languages should be translated into local languages of the target user communities. 

The Globally Harmonized System (GHS) for classification and labelling of chemicals hazard communication system advocates for warning messages to contain features that are known or easily comprehended and not too abstract to the users [57]. From Figure 1, 34.6% of the respondents did not read instructions because of being illiterate and the use of foreign language would benefit from Uganda adopting the GHS tool for safe use of pesticides. They would be able to comprehend symbols and pictograms, the core tools of the GHS hazard communication system [58, 59]. Respondents suggested that better pesticide labeling would increase cautionary behaviors among the users. 

Some respondents (21.2%) did not have any specific reason for not reading the instructions prior to use. Grey and colleagues [51] in UK reported that about 42.5% of indoor pesticide users did not read product instructions. In the present survey, 52% did not read for the various reasons as indicated in Figure 1.

In many developing countries like Uganda, legislation, monitoring, and enforcement regarding indoor pesticides use are inadequate [8]. Some pesticides that are either of severely restricted use or been banned globally due to their adverse health effects are still being marketed and used, for example, diazinon, a highly toxic organophosphate [60]. In this study, diazinon was one of the pesticides still being used for indoor pest control. 

Efforts should be made to ensure that pesticide users get to read or be explained to and understand the instructions for application so as to reduce health effects associated with improper use of pesticides. This can effectively be done at the point of sale, where users can interact with the pesticide seller for in-depth explanations on the use of the product being purchased. This approach would need to extend and to make more comprehensive the training required for all pesticide sellers in the country, and particularly in the urban areas. Product labeling containing hazard information and instruction for use should be enforced and in languages understood by user communities.

#### 3.5.2. Time and Frequency of Use of In-Home Pesticides

The time when pesticides were applied varied, with 52% respondents doing it at night and 49% during the day mainly to keep away houseflies. Those that applied pesticides daily were 40%, once a week 31%, and 15% applied once a month. Frequent application if not done correctly in the right places and quantities is likely to pose health risks. Those that applied once a month were mostly households that utilized professional fumigators to do the spraying. Others (14%) applied whenever pest infestation was high, for example, against mosquitoes during rainy seasons. 

The frequency of pesticide application could be reduced by observing proper domestic solid waste management and reducing mosquito breeding places. 

Limitations of the study to a degree included recall bias among the respondents concerning the pesticide names and precisely how they were used. There is information bias as some respondents did not want to reveal some pests in their homes especially lice and bedbugs since both of these would be associated with extremely poor hygiene.

## 4. Conclusion

Mosquitoes were the most prevalent of pests in the study area. 

Since Uganda is endemic to malaria, insecticide use is widespread and likely to increase. Consequently there is increased health risks associated with insecticide use in poor housing conditions.

Households employed various methods for pest control, with pesticide application being the highest prevalent method.

Less than half of the respondents read the manufacturers' instructions before use of the pesticides in their homes. It is important that instructions are in a language that can be read and understood and consequently translated into good practice in the application of pesticides. Governments in low-income countries like Uganda should ensure that such provisions of the GHS hazard communication system exist for the health and safety of their nationals by adopting the system. 

Health inspectors whose work includes advising communities on the best ways to apply pesticides were the least people relied upon for that information, with friends and sales points being most relied upon for this information. It is recommended that sales points should be formalized as places where pesticide users obtain information on proper pesticide use.

## Figures and Tables

**Table 1:** Demographic characteristics of participants.

Characteristic	Frequency^a^	%^a^
Respondent gender		
Female	71	71
Male	29	29
Age		
15–29	33	34.7
30–44	31	32.6
45-above	31	32.6
Educational level		
Tertiary	20	20
Secondary	33	33
Primary	23	23
None	24	24
Marital status		
Single	29	29
Married	46	46
Separated	15	15
Widowed	10	10

^a^Totals may not be adding to 100 households or to 100% owing to some nonresponses to survey questions.

**Table 2:** Pests present in homes of Kireka ward.

Type of pest	Prevalence of response among all respondents (*n* = 100) %	Prevalence of response among all responses given (*n*=256)^b^ %
Mosquitoes	83	32.4
Cockroaches	69	27
Rats	52	20.3
Bedbugs	34	13.3
Lice	7	2.7
Others^a^	11	4.3

^a^Responses included ants, bats, fleas, termites, ticks.

^b^Multiple responses were given.

**Table 3:** Pest control practices employed in Kireka ward and source of information for choice of insecticide.

Practiced pest control method	Prevalence of response among respondents (%)
Rat glue and traps	22
Proper garbage disposal	34
Biological control	20
Pesticides application	98
*Form of insecticide*	
Powder and concentrate	4.1
Insecticide chalks	39.8
Coils	38.8
Sprays	71.4
Wettable powders	41.8
*Source of information for choice of insecticide*	
Health inspectors	24.5
Sales points	48
Advertisements	33.7
Friends	53.1
